# First human cell-based cultivation system for the syphilis spirochete *Treponema pallidum*

**DOI:** 10.1186/s12866-026-04856-5

**Published:** 2026-02-24

**Authors:** Juraj Bosák, Matěj Hrala, Petra Pospíšilová, Michaela Bosakova, David Šmajs

**Affiliations:** 1 Department of Biology, Faculty of Medicine, Masaryk University, Brno, Czech Republic; 2 Institute of Animal Physiology and Genetics of the CAS, Brno, Czech Republic

**Keywords:** Treponema, T. pallidum, syphilis, in vitro, human foreskin fibroblasts

## Abstract

**Background:**

*Treponema pallidum* subsp. *pallidum* (*T. pallidum*), the etiological agent of syphilis, was first isolated from infected humans in the early 20th century and, for decades, could only be propagated in laboratory rabbits. In 2018, a reproducible in vitro cultivation system was established using rabbit epithelial Sf1Ep cells and a complex culture medium, allowing stable growth of *T. pallidum*. While in vitro cultivation has revolutionized *T. pallidum* research, further humanization of this system is necessary to study host–pathogen interactions.

**Results:**

We evaluated three human foreskin fibroblast cell lines (HFF1, HFFC, and MoNa) for their ability to support in vitro growth of *T. pallidum* as an alternative to rabbit Sf1Ep cells. All human cell lines displayed typical fibroblast morphology and showed growth rates comparable to or slower than Sf1Ep cells, a feature favorable for treponemal propagation. Eight available *T. pallidum* strains representing the Nichols-like (DAL-1, Madras, London, Haiti B) and the SS14-like clusters (Mexico A, SS14, Grady, Philadelphia 1) sustained growth on human fibroblast cells for ten weeks. Notably, DAL-1 cultures have been continuously maintained on human fibroblasts for over one year. Cultivation on human fibroblasts resulted in slower overall growth of *T. pallidum* compared to rabbit cells. Nevertheless, Nichols-like strains retained their higher replication rates relative to SS14-like strains, consistent with observations from rabbit-based systems.

**Conclusions:**

Human foreskin fibroblasts can serve as effective cells for support of *T. pallidum* growth in vitro. This approach represents a step toward humanized culture conditions, enabling further investigation of the pathogenesis of syphilis.

**Supplementary Information:**

The online version contains supplementary material available at 10.1186/s12866-026-04856-5.

## Background

*Treponema pallidum* subsp. *pallidum* (*T. pallidum*), the causative agent of syphilis, is a global health threat. Although effective antibiotic treatment is available, the number of syphilis cases has increased in recent years worldwide [1, 2], reaching an estimated 8 million new infections in 2022 [3].

*T. pallidum* was successfully isolated and propagated in laboratory rabbits since the early 20th century; despite numerous efforts [4], it remained uncultivable in vitro for years. In 1981, an in vitro cultivation system was introduced, taking advantage of rabbit epithelial Sf1Ep cells, which allowed multiplication of *T. pallidum* for up to 12 days [5]. Only in 2018, long-term cultivation of *T. pallidum* was achieved [6], by complementing the Sf1Ep cells with a low-oxygen atmosphere and TpCM-2 medium, along with weekly subcultures. Several *T. pallidum* subsp. *pallidum* strains (Nichols, DAL-1, Madras, Haiti B, SS14, Mexico A, Philadelphia 1, Grady, UW231B, UW249B) and the *T. pallidum* subsp. *endemicum* strain Bosnia A have been successfully cultivated in vitro [6–9]. Furthermore, the first in vitro isolation and propagation of six contemporary strains (MU1-6) directly from syphilis patients was achieved in 2025 [10].

 In vitro cultivation has revolutionized *T. pallidum* research, providing a platform for investigating treponemal physiology [8, 9], antibiotic susceptibility [11–15], and genetic manipulation [15, 16]. However, humanization of this culture system is necessary to study the host-pathogen interaction. To overcome this limitation, we evaluated the capacity of three cell lines derived from human foreskin to support *T. pallidum* growth in vitro. Human foreskin fibroblasts have been used as feeder cells for maintenance of human pluripotent stem cells [17].

## Methods

### *T. pallidum* strains and routine in vitro cultivation

*T. pallidum* strains Haiti B, Madras, Philadelphia 1, and Grady were kindly provided by Dr. D. Cox (Centers for Disease Control and Prevention, Atlanta, USA). *T. pallidum* strains SS14 and Mexico A were kindly provided by Dr. K. Hawley (University of Connecticut School of Medicine, Farmington, USA). *T. pallidum* strain DAL-1 (internally marked as PV171) was kindly provided by Dr. D. Edmondson (University of Texas Health Science Center, Houston, USA). *T. pallidum* strain London (allelic profile 10.14.10), isolated in the 1970s, was purchased from Pettingill Technology (Oxford, UK). All *T. pallidum* strains were provided as frozen suspensions from rabbit testes with unknown concentrations of treponemal cells.

In our laboratory, all *T. pallidum* strains have been continuously cultivated in vitro for over two years [8]. Briefly, treponemes were cultivated in well (triplicate in a 6-well plate 92406, Techno Plastic Products, Switzerland) containing TpCM-2 medium (4 mL) and a monolayer of rabbit Sf1Ep cells (50,000 cells). Every week, the treponemal cultures were harvested (2 × 500 µL, Trypsin-EDTA, 37 °C, 5 min), centrifuged to deplete Sf1Ep cells (250 × g, 5 min), and the supernatant containing treponemes (typically 250–1000 µL) was inoculated into a freshly prepared well. The *T. pallidum* cultures were cultivated at 34 °C in a low oxygen atmosphere (2.5% O_2_, 5.0% CO_2_, balanced by N_2_).

During subculturing, treponemal numbers and viability (i.e., motility) were investigated using dark-field microscopy. Briefly, each treponemal culture (5 µL) was examined using a BX53 microscope (Olympus Corporation, Tokyo, Japan) at 400× magnification, where five individual fields were screened. The number of treponemes was estimated using the following formula: one treponeme per field equals 340,000 treponemes per mL of culture [8].

### Supporting cells and routine in vitro cultivation

Rabbit Sf1Ep cells (NBL-11) were kindly provided by Dr. D. Edmondson (UT Health Science Center, Houston, USA) and were originally purchased from the American Type Culture Collection (Rockville, MD, USA; ATCC line CCL-68). Cultivation of rabbit Sf1Ep cells was performed as described previously [6]. Briefly, rabbit cells were grown in Sf1Ep medium consisting of Eagle’s MEM (M4655, Sigma-Aldrich) supplemented with non-essential amino acids (1%; M7145, Sigma-Aldrich), L-glutamine (1%; G7513, Sigma-Aldrich), sodium pyruvate (1%; S8636, Sigma-Aldrich), and heat-inactivated fetal bovine serum (10%; F4135, Sigma-Aldrich). Cultures were maintained at 37 °C in an atmosphere with 5% CO₂ and subcultured weekly (750,000 cells, T75 plastic flask, Techno Plastic Products, Switzerland). In this study, Sf1Ep cells with passage numbers ranging from 40 to 60 were employed as supporting cells for *T. pallidum* growth in vitro.

Three distinct human foreskin fibroblast cell lines were used in this study. Human foreskin fibroblasts HFF1 (SCRC-1041; ATCC) and HFFC (300715; Cytion) were purchased, while the third cell line (MoNa) was kindly provided by Dr. Vladimir Rotrekl (Masaryk University), and was originally obtained from the National Tissue Centre (Czech Republic). HFF1 and MoNa cells were grown in DMEM medium (31966-021; Gibco) supplemented with non-essential amino acids (1%; M7145, Sigma-Aldrich) and heat-inactivated fetal bovine serum (10%; F4135, Sigma-Aldrich). HFFC cells were grown in DMEM/F12 medium (LM-D1222, Biosera) supplemented with insulin (10 µg/L; 3435, R&D Systems) and FGF2 (10 µg/L; 233-FB, R&D Systems), according to the manufacturer’s recommendations. All human foreskin fibroblast cell lines were maintained at 37 °C in an atmosphere with 5% CO_2_ and subcultured weekly (750,000 cells, T75 plastic flask, Techno Plastic Products, Switzerland). During subculturing, cell morphology was examined using a CKX53 microscope (Olympus Corporation, Tokyo, Japan), and cell numbers were quantified with an EVEPlus cell counter (NanoEnTek, Seoul, Korea).

While the rabbit cell line is immortalized [6, 7], the used foreskin fibroblasts are primary cell lines and require initiation from a frozen vial every 15–20 weeks.

### Long-term cultivation of *T. pallidum* with individual human foreskin cell lines

For two *T. pallidum* strains, DAL-1 and SS14, in vitro cultivation with individual human foreskin cell lines was performed. The cryopreserved *T. pallidum* cultures (i.e., stored at -80 °C in 15% glycerol) were thawed and inoculated (500 µL/well) into cultivation wells for each human foreskin fibroblast cell line: HFF1, HFFC, and MoNa (50,000 cells/well). For weekly subcultures, a single aliquot (250–1000 µL) of *T. pallidum* culture was transferred from each well to a fresh well. Other in vitro cultivation parameters were identical to the previously published protocol (see above). Every subculture, the number of treponemes was quantified using dark-field microscopy (400× magnification, 5 fields of view) as described above. In vitro cultures of SS14 strain were intentionally discontinued after 10 subcultures, whereas DAL-1 in vitro cultures remain ongoing (currently over one year).

### Quantification of differences in *T. pallidum* growth support among human foreskin cell lines

To quantify the ability of individual human foreskin fibroblast cell lines to support *T. pallidum* growth, an in vitro culture of strain DAL-1 was adjusted to a standardized inoculation dose (10^6^ treponemes/well) and simultaneously cultivated on individual cell lines (HFF1, HFFC, MoNa, and rabbit Sf1EP) in triplicates. This parallel setup was repeated five times, resulting in a total of 15 biological replicates per condition. Other in vitro cultivation parameters were identical as in the long-term culture. Following a seven-day cultivation period, treponemes were harvested (2 × 500 µL Trypsin/EDTA) and numbers of treponemes were quantified using dark-field microscopy (400× magnification, 10 fields of view) as described above.

### Cultivation of eight *T. pallidum* strains using mixed human foreskin cell lines

For in vitro cultivation with human foreskin fibroblasts, in vitro cultures of *T. pallidum* strains DAL-1, London, Haiti B, Madras, SS14, Mexico A, Philadelphia 1, and Grady were used. The cryopreserved *T. pallidum* cultures (i.e., stored at -80 °C in 15% glycerol) were thawed and inoculated (500 µL) into cultivation wells containing an equal mixture of three human foreskin fibroblast cell lines (HFF1/HFFC/MoNa − 50,000 cells/well). Since the limited availability of primary fibroblast stocks precluded the simultaneous cultivation of all *T. pallidum* strains on individual cell lines, a mixture of supporting cells was used. *T. pallidum* strains were subcultured weekly by transferring 1 mL of culture to a fresh well. Other in vitro cultivation parameters were identical as in the long-term culture. Every subculture, the numbers of treponemes were quantified using dark-field microscopy (400× magnification, 5 fields of view) as described above. In vitro cultivation of *T. pallidum* strains was performed for 10 subcultures (70 days).

### PCR detection of rabbit DNA

To verify the absence of residual rabbit cells in the cultures, the presence of rabbit DNA was assessed by PCR targeting the ATP7A gene, which encodes the copper-transporting P-type ATPase. Briefly, an aliquot of the *T. pallidum* in vitro culture was collected (200 µL), and a total DNA was isolated using the QIAamp DNA Blood Mini Kit (Qiagen, Hilden, Germany) according to the blood and body fluid protocol, with a final elution volume of 100 µL. PCR amplification was performed using the primers 5´-GCGTCTGAAGAACACACCAG-3´ and 5´-GGGGAACTCTCAGCTTCACT-3´, yielding a 306 bp amplicon. Each PCR reaction (final volume: 25 µL) consisted of nuclease-free water (7.3 µL), 2.5 mM dNTP mixture (2 µL), 5× PrimeSTAR GXL buffer (5 µL), 0.4 µM primers (0.1 µL each), PrimeSTAR GXL polymerase (0.5 µL; Takara Bio Europe, France), and isolated DNA (10 µL). PCR cycling conditions were as follows: initial denaturation at 94 °C (1 min); 8 cycles of 98 °C (10 s), 68 °C (15 s; with a decrease of 1.0 °C per cycle), and 68 °C (1 min 45 s); followed by 35 cycles of 98 °C (10 s), 61 °C (15 s), and 68 °C (1 min 45 s); with a final extension at 68 °C (7 min).

## Results and discussion

**Fig. 1:**
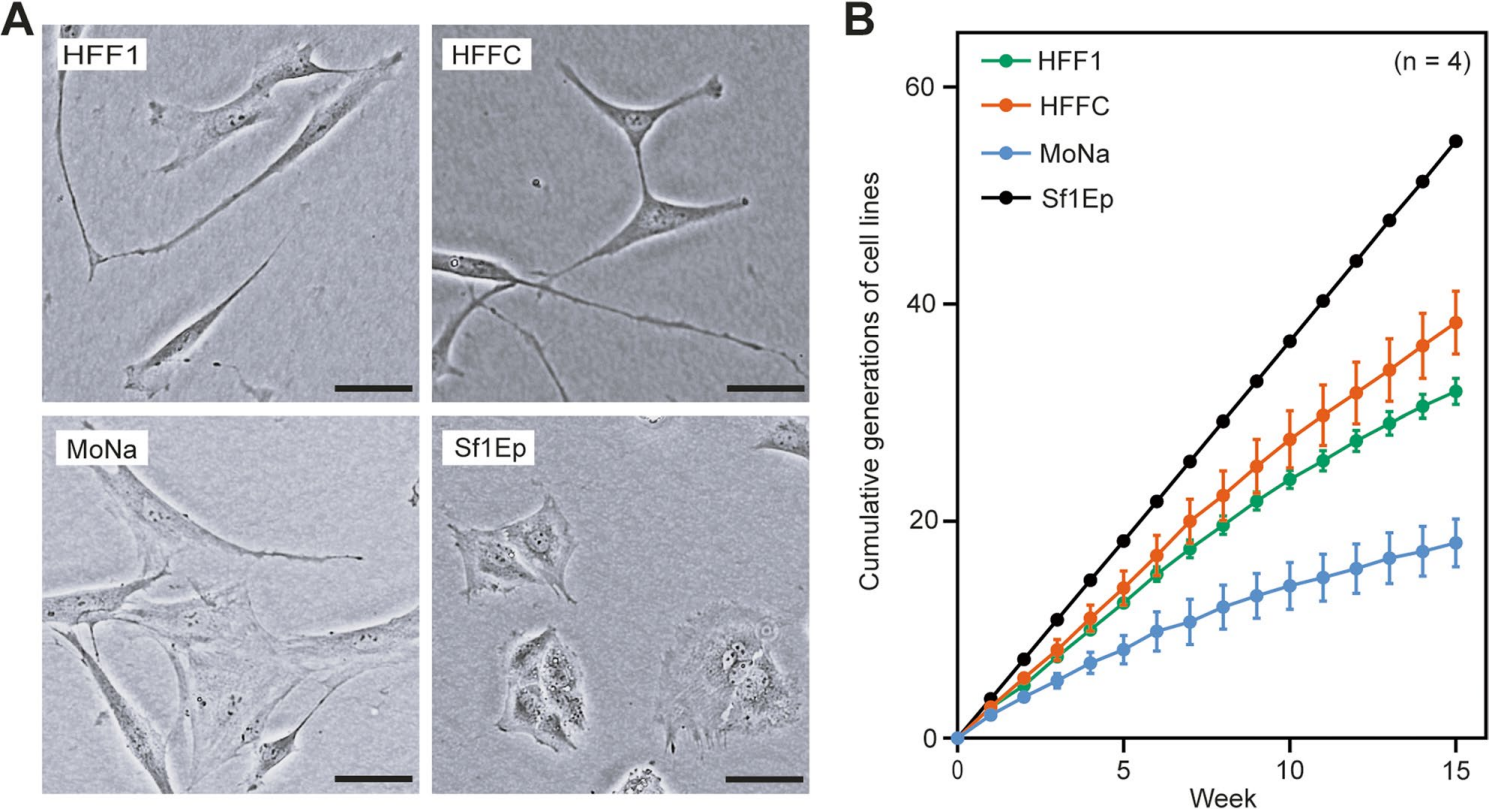
Comparison of human foreskin fibroblast cell lines (HFF1, HFFC, MoNa) and rabbit Sf1Ep cells. All three foreskin cell lines have fibroblast morphology (**A) **and lower multiplication compared to rabbit epithelial Sf1Ep cells (**B)**. The growth rate of the human foreskin fibroblast cell lines is presented as cumulative generations calculated over fifteen weekly subcultures. Each data point represents the mean (± standard error of the mean) from four separate experiments (*n* = 4). Scale bars, 50 μm

We tested three available human foreskin cell lines - HFF1, HFFC, and MoNa. All three cell lines showed fibroblast morphology (Fig. 1A), which was different from the rabbit epithelial Sf1Ep cells used in the original in vitro cultivation system. The growth rate of the human foreskin fibroblast cell lines under the same culture protocol (i.e., weekly inoculation of 750,000 cells into T75 flask) was comparable to or slower than the rabbit Sf1Ep cells (Fig. 1B). While the rabbit cell line is immortalized [6, 7] and multiplies uniformly across subcultures with an average generation time of 45.56 h (± 0.23), the foreskin fibroblasts used in this study are senescence-prone primary cell lines. According to previous studies, the use of a slow-growing cell line for *T. pallidum* co-culture appears crucial for successful treponemal cultivation [6].

**Fig. 2:**
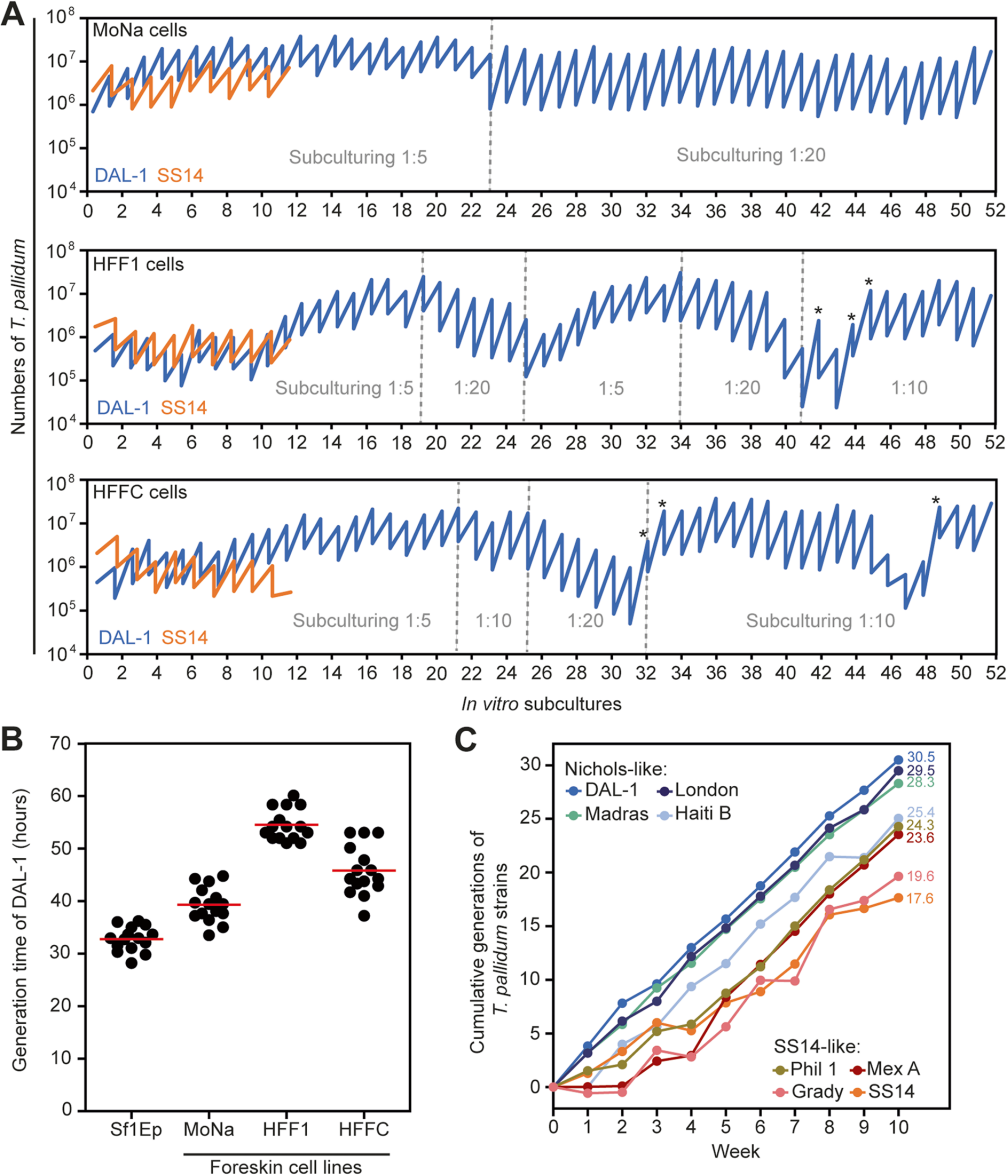
Human foreskin fibroblast cell lines support the growth of *T. pallidum *in vitro*. ***A **Human foreskin fibroblast cell lines (MoNa, HFF1, and HFFC) support the growth of *T. pallidum* in long-term, continuous, in vitro cultivation. While cultivation of strain SS14 was discontinued after 10 weeks, in vitro cultures of the DAL-1 strain have been ongoing for over one year. During this period, scheme of subcultures was optimized (up to 1:20). A booster passage (*), extending the standard 7-day subculture to a 14-day period with a cultivation medium exchange on day 7, was employed to enrich the treponemal culture during critical declines. In vitro cultivation was performed in triplicate (i.e., in three cultivation wells) and the mean values for each subculture are presented in graph. Source data from individual wells are shown in Supplementary Table 1. **B **Parallel cultivation for seven days (*n* = 15) with defined DAL-1 inoculum (10^6^ treponemes) validated the differences in growth support of individual foreskin cell lines. Treponemal growth on human foreskin fibroblast cells was slower compared to growth on rabbit epithelial Sf1Ep cells. Red bar, mean. **C **Human foreskin-based cultivation system supports the growth of various *T. pallidum* strains (*n* = 8), from the Nichols-like as well as the SS14-like cluster. Note that human foreskin fibroblast cells were prepared as an equal mixture of three tested cell lines. Each *T. pallidum* strain was cultivated in a single in vitro well, representing a sole experimental replicate used for data acquisition

Next, all three human foreskin fibroblast cell lines were used to cultivate *T. pallidum *in vitro, employing a protocol similar to that previously published for rabbit cells [6]. We inoculated strains DAL-1 and SS14 (representing two genomic clusters [18]) from cryopreserved in vitro cultures into wells with individual human foreskin fibroblast cell lines. Using 5× dilution scheme during weekly subcultures (i.e., 1 mL inoculum, see Material and Methods), all three human foreskin cell lines were able to support the continuous cultivation of both *T. pallidum* strains. While cultivation of the SS14 strain was discontinued after 10 weeks due to limited resources, the cultivation of DAL-1 is still ongoing (over one year) (Fig. 2A). During long-term maintenance of DAL-1 strain, stable in vitro cultures were achieved with weekly 1:20 subcultures (250 µL inoculum) on MoNa cells and 1:10 subcultures (500 µL inoculum) on HFF1 and HFFC cells.

For precise quantification of human foreskin cells’ growth support, we performed a 7-day parallel cultivation of *T. pallidum* DAL-1 with identical inoculation dose (10^6^ treponemes) to minimize subculture variability during long-term maintenance. Compared to the average generation time of strain DAL-1 on rabbit cells (32.8 h), growth on foreskin cells was slower: 39.3 h on MoNa cells, 45.8 h on HFFC cells, and 54.5 h on HFF1 cells (Fig. 2B). 

The overall lower treponemal growth observed on foreskin fibroblasts is in accordance with the study of Fieldsteel et al. (1979), who reported that Sf1Ep cells offered the best support for *T. pallidum* compared to other tested cell lines, including human foreskin cells [19]. However, further optimization of the culture conditions, such as adjusting the medium composition or the seeding density of the supporting fibroblasts, could potentially improve treponemal yields. 

The differences in growth support among the human foreskin cell lines likely result from their intrinsic biological characteristics. For instance, primary fibroblast lines may differ in their differentiation states or metabolic profiles, which could result in the observed variations in treponemal yields. Although this aspect requires further investigation, our results indicate that the capacity to support *T. pallidum* multiplication is likely a general characteristic of human foreskin fibroblasts. 

To evaluate the overall ability of the human foreskin fibroblast to support growth of a broad spectrum of *T. pallidum* strains, eight available strains (DAL-1, Madras, Haiti B, London, SS14, Mexico A, Philadelphia 1, and Grady) were cultured with a mixture of all three human cell lines (1:1:1 ratio of MoNa, HFFC and HFF1; see Material and Methods). Through the 10-week culture, the human foreskin fibroblasts successfully supported the growth of all tested *T. pallidum* strains (Fig. 2C). The estimated generation time of 50.5 h for the DAL-1 strain is consistent with the range observed across individual human cell lines (39.3–54.5 h; Fig. 2B), suggesting that the cell mixture had no substantial effect on treponemal proliferation. As expected, other *T. pallidum* strains exhibited slower growth rates. Notably, most of the Nichols-like strains grew faster than the SS14-like strains, achieving approximately 30 versus 20 generations, respectively. This finding aligns with the observations in rabbit co-cultures [7, 8], and thus confirms that the human cell model successfully reproduces the previously found biological differences between *T. pallidum* strains. 

The residual rabbit cells from the *T. pallidum* inocula could theoretically be responsible for partial treponemal growth; however, their presence in the cultures after several passages is unlikely. The cryostorage of inocula in glycerol was used to eliminate 99.99% of the rabbit cells, leaving only 41.7 ± 10.14 residual cells per well (mean ± SEM, n = 6[]). Such a minimal count is insufficient to support treponemal growth in vitro [7] and it was further reduced by progressive dilution during subculturing (calculated as 15-fold rabbit cell multiplication vs. 50-fold dilution in each passage). Finally, PCR screening verified a rabbit-free system at all tested time points (see Supplementary Fig. S1).

Despite their widespread use for *T. pallidum* propagation, the rabbit-based models (in vitro and in vivo) may be inadequate for identifying virulence factors or for fully understanding of syphilis pathogenesis. Rabbits are susceptible to *T. paraluisleporidarum* ecovar Cuniculus and Lepus, which possess numerous inactivated genes compared to *T. pallidum* [20, 21]. Furthermore, since the rabbit treponemes are non-infectious to humans [22], significant differences in *T. pallidum* interaction with human versus rabbit cells are expected.

Taken together, this study presents the first stable system for the cultivation of *T. pallidum* strains with human cells in vitro. Although this human-cell model currently exhibits slower treponemal growth than traditional rabbit-based system, it represents a more relevant tool for studying interactions between the syphilis agent and its human host. The observed differences in growth rates are likely due to factors such as cell type, species origin, and cell multiplication rates. Despite these current limitations, our system provides a valuable alternative for syphilis research. Future efforts should focus on optimizing cultivation schemes and exploring other human cell types, such as epithelial and endothelial lines, to further expand our understanding of syphilis pathogenesis.

## Conclusions

In this work, we introduced human foreskin fibroblasts as supporting cells for *T. pallidum* cultivation in vitro. This represents a next step to humanization of *T. pallidum* culture conditions that could help elucidate the pathogenesis of syphilis.

## Supplementary Information

Supplementary Material 1.

Supplementary Material 2.

## Data Availability

The datasets used and/or analyzed during the current study are available from the corresponding author on reasonable request (D.Š., dsmajs@med.muni.cz).
