# Supplementary material for: First human cell-based cultivation system for the syphilis spirochete Treponema pallidum
Source: BMC Microbiol. 2026 Feb 24;26:288. doi: 10.1186/s12866-026-04856-5 (PMC13037089; doi:10.1186/s12866-026-04856-5)
Supplement: Supplementary file 1 — Supplementary Material 1. [file 12866_2026_4856_MOESM1_ESM.pdf]

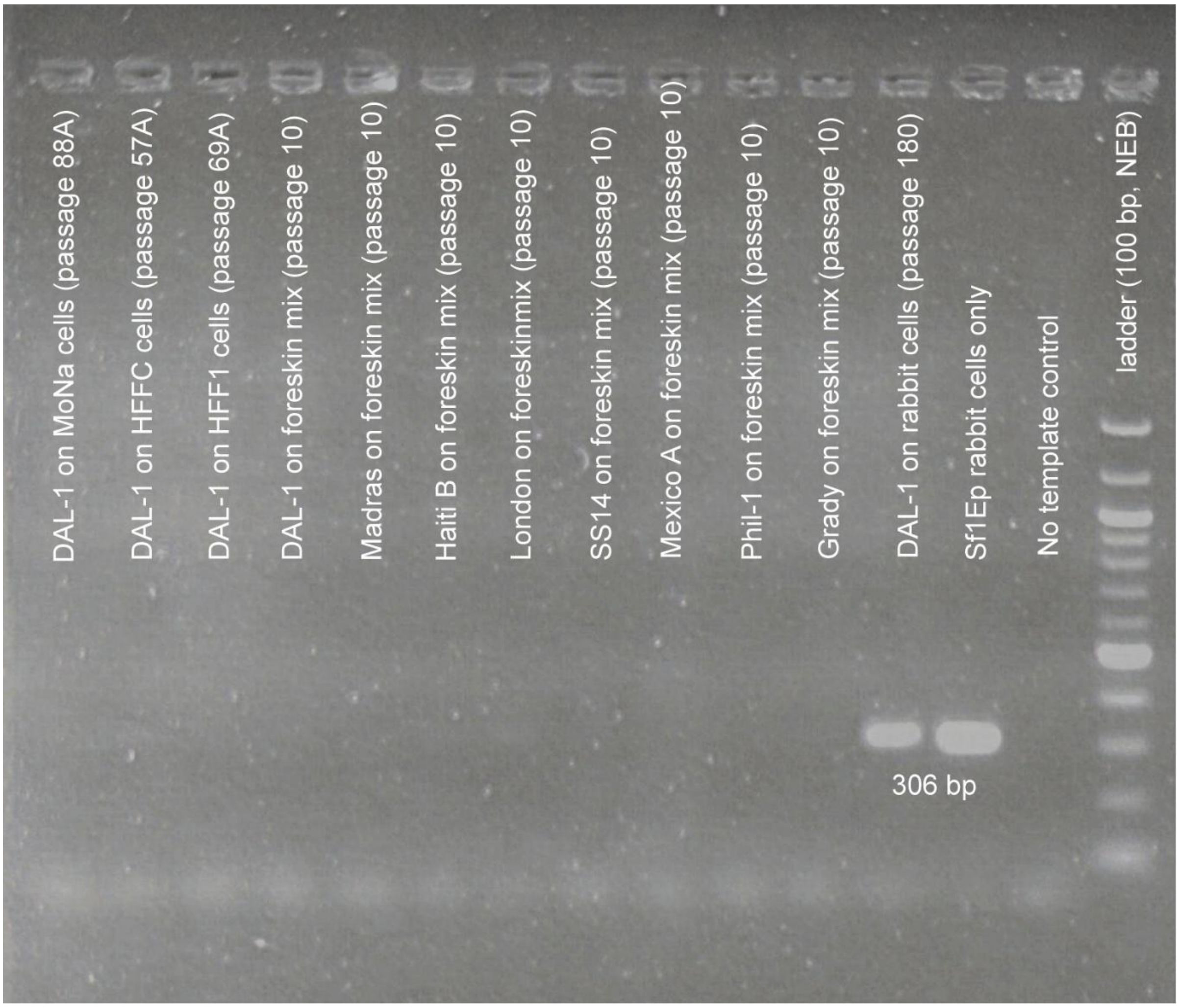

Figure S1. PCR detection of rabbit cells in humanized *in vitro* cultures. During the cultivation of *T. pallidum* with human foreskin fibroblasts, an *in vitro* cultures were repeatedly tested for the presence of rabbit cells (based on the ATP7A gene detection, PCR product length 306 bp). A representative gel image is shown. All tested time points were negative for rabbit DNA. PCR products were visualized using 1.5% agarose gel electrophoresis (running conditions: 90V, 70 min) and the product size was evaluated using a 100 bp DNA ladder (N3231; New England Biolabs).
